# Trisubstituted Pyrazolopyrimidines as Novel Angiogenesis Inhibitors

**DOI:** 10.1371/journal.pone.0054607

**Published:** 2013-01-15

**Authors:** Sabine B. Weitensteiner, Johanna Liebl, Vladimir Krystof, Libor Havlíček, Tomáš Gucký, Miroslav Strnad, Robert Fürst, Angelika M. Vollmar, Stefan Zahler

**Affiliations:** 1 Department of Pharmacy, Ludwig-Maximilians-University, Munich, Germany; 2 Laboratory of Growth Regulators, Faculty of Science, Palacký University and Institute of Experimental Botany AS CR, Olomouc, Czech Republic; 3 Isotope Laboratory, Institute of Experimental Botany AS CR, Prague, Czech Republic; 4 Centre of the Region Haná for Biotechnological and Agricultural Research, Faculty of Science, Department of Growth Regulators, Palacký University, Olomouc, Czech Republic; University of Chicago, Department of Medicine, United States of America

## Abstract

Current inhibitors of angiogenesis comprise either therapeutic antibodies (e.g. bevacicumab binding to VEGF-A) or small molecular inhibitors of receptor tyrosin kinases like e.g. sunitinib, which inhibits PDGFR and VEGFR. We have recently identified cyclin-dependent kinase 5 (Cdk5) as novel alternative and pharmacologically accessible target in the context of angiogenesis. In the present work we demonstrate that trisubstituted pyrazolo[4,3-*d*]pyrimidines constitute a novel class of compounds which potently inhibit angiogenesis. All seven tested compounds inhibited endothelial cell proliferation with IC_50_ values between 1 and 18 µM. Interestingly, this seems not to be due to cytotoxicity, since none of them showed acute cytotoxic effects on endothelial cells at a concentration of 10 µM,. The three most potent compounds (LGR1404, LGR1406 and LGR1407) also inhibited cell migration (by 27, 51 and 31%, resp.), chemotaxis (by 50, 70 and 60% in accumulative distance, resp.), and tube formation (by 25, 60 and 30% of total tube length, resp.) at the non-toxic concentration of 10 µM. Furthermore, angiogenesis was reduced *in vivo* in the CAM assay by these three compounds. A kinase selectivity profiling revealed that the compounds prevalently inhibit Cdk2, Cdk5 and Cdk9. The phenotype of the migrating cells (reduced formation of lamellipodia, loss of Rac-1 translocation to the membrane) resembles the previously described effects of silencing of Cdk5 in endothelial cells. We conclude that especially LGR1406 and LGR1407 are highly attractive anti-angiogenic compounds, whose effects seem to largely depend on their Cdk5 inhibiting properties.

## Introduction

Angiogenesis, the sprouting of new vessels from the existing vasculature, mainly occurs during embryonic development and growth. In the adult it is restricted to distinct physiological processes, e.g. wound healing, by a balance of pro- and anti-angiogenic factors [1]. Unregulated angiogenesis is one of the hallmarks of cancer [2]. Tumor growth is highly dependent on proper supply with oxygen and nutrients and removal of metabolic waste. Therefore, angiogenesis is crucial for tumor survival and proliferation, and tumor size remains limited unless the tumor switches to an angiogenic phenotype [3]. The intent to stop tumor growth and finally starve the tumor by disrupting angiogenic signaling has led to the development of anti-angiogenic drugs for anticancer therapy. Agents addressing vascular endothelial growth factor (VEGF) induced angiogenesis have already been successfully introduced into tumor therapy [4].

However, in clinical use it has become apparent that anti-angiogenic tumor therapy is more challenging than expected: Many tumors are refractory to VEGF-blockade or become resistant during treatment. This evasive resistance [5] can be caused by a shift to alternative angiogenic signaling pathways due to a pre-existing multiplicity of redundant pro-angiogenic signals. Therefore novel targets in angiogenesis need to be identified and characterized as a basis for future therapeutic concepts.

Cdk5 has been discovered as a neuronal cdc2-like serine/threonine kinase (nclk) in 1992 [6], [7]. Despite its high sequence homology with the mitotic Cdk1 (cdc2), Cdk5 is not involved in cell cycle control and unique among the Cdks in its regulation and function. On the cellular level, Cdk5 is well-described in neurons as the key hub in the dynamic network of trafficking and transport, integrating signals in cytoskeletal dynamics during neuronal migration, in synaptic plasticity and synaptic vesicle endo- and exocytosis, cell adhesion and axon guidance, neuromuscular development and pain signaling [8], [9]. Although Cdk5 expression and activity is highest in the central nervous system [6], Cdk5 is also expressed in various tissues, and an increasing body of research uncovers extraneuronal functions of Cdk5, where it is involved in the regulation of migration, cell death and survival, glucose metabolism and inflammation [10], [11].

(*R*)-roscovitine or CYC-202/seliciclib – in the following referred to as roscovitine – belongs to the class of 2,6,9-trisubstituted purines. It is one of the best-known Cdk inhibitors [12], and is currently tested in several phase I and phase II clinical trials for tumor treatment [13]. Roscovitine inhibits mainly Cdk1, Cdk2, Cdk5, Cdk7 and Cdk9 and exerts anti-mitotic and pro-apoptotic effects in a wide range of tumor cells [14]. Cell-cycle independent actions of roscovitine mainly derive from Cdk5 inhibition and include anti-angiogenic [15] and anti-inflammatory [16] effects, inhibition of cell migration and motility [17], [18] and modulation of glucose metabolism [19].

Anti-angiogenic actions of Cdk inhibitors have been observed *in vitro* and *in vivo* [20], [21], [22]. Recently, we have demonstrated that the anti-angiogenic effect of roscovitine most likely results from impaired endothelial cell migration. The effect on migration was traced down to Cdk5 inhibition which led to Rac1 inactivation and lamellipodia disruption [15]. A promising novel strategy in anti-angiogenic therapy may, therefore, be inhibition of Cdk5. To date, improved Cdk inhibitors have mainly been developed in order to block cancer cell proliferation but have not systematically been optimized and evaluated for anti-angiogenic action. Therefore, the aim of the present study was to evaluate the *in vitro* and *in vivo* anti-angiogenic potency of newly prepared roscovitine-derived Cdk inhibitors built on the pyrazolo[4,3-*d*]pyrimidine heterocyclic core.

## Results

### Newly prepared pyrazolo[4,3-*d*]pyrimidine inhibitors of Cdks

**Figure 1:**
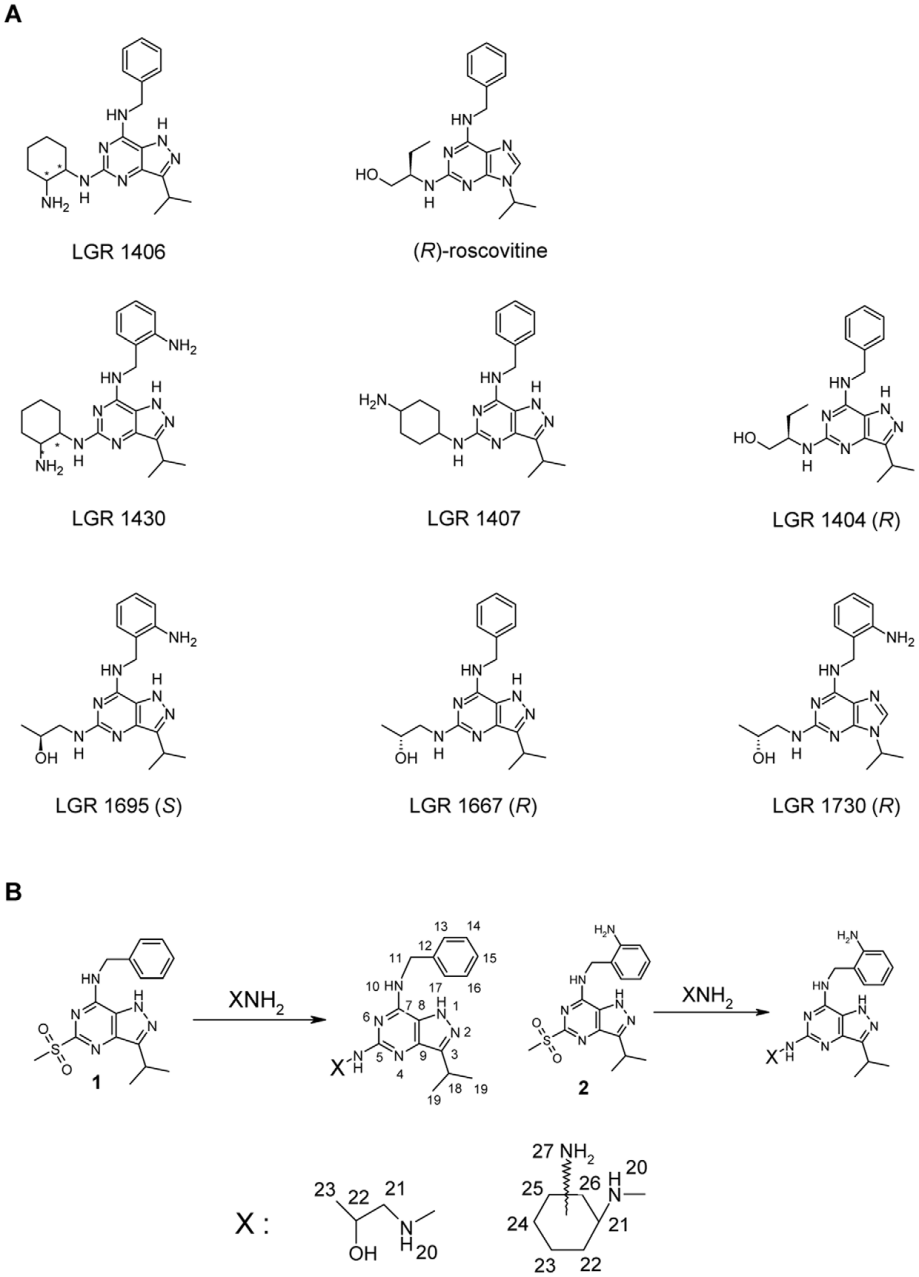
Chemical structures and synthesis of tested compounds in comparison to (*R*)-roscovitine. A: The LGR compounds have been modified in comparison to (*R*)-roscovitine in one or more of the following aspects: 1) The purine scaffold has been changed to pyrazolo[4,3-*d*]pyrimidine (LGR 1404, 1406, 1407, 1430, 1667, 1695), with LGR 1404 being a bioisoster of (*R*)-roscovitine. 2) In the aminobenzyl group, an additional ortho amino function is present. (LGR 1430, 1695, 1730). 3) The residue at purine C2 or pyrazolo[4,3-*d*]pyrimidine C5 respectively, differs from (*R*)-roscovitine either in structure and/or stereochemistry. Defined configurations are shown in the chemical structures. LGR 1407 contains no stereocenter. LGR 1406 and LGR 1430 are an equal mixture of 4 stereoisomers: the trans enantiomers (*R, S*) and (*S, R*); and the cis enantiomers (*R, R*) and (*S, S*). B: The synthesis of new compounds and numbering of atoms for NMR spectra.

We have recently prepared 7-benzylamino-5(*R*)-[2-(hydroxymethyl)-propyl]amino-3-isopropyl-1(2)*H*-pyrazolo[4,3-*d*]pyrimidine (compound LGR1404) as a bioisostere of the well-known Cdk inhibitor roscovitine [23]. Our experiments proved that it is a more potent Cdk inhibitor and also its anticancer activity *in vitro* exceeds that of roscovitine. Therefore, and based on our knowledge of structure-activity relationships for related purine Cdk inhibitors, we have prepared a set of new and potent Cdk inhibitors with the pyrazolo[4,3-*d*]pyrimidine scaffold (Figure 1A). Details on the synthetic route are described in the Material and Methods section and Figure 1B. The new compounds have been modified in comparison to (*R*)-roscovitine in one or more of the following aspects: 1) The purine scaffold has been changed to pyrazolo[4,3-*d*]pyrimidine (LGR 1404, 1406, 1407, 1430, 1667, 1695). 2) In the aminobenzyl group, an additional ortho amino function is present (LGR 1430, 1695, 1730). 3) The residue at purine C2 or pyrazolo[4,3-*d*]pyrimidine C5 differs from (*R*)-roscovitine either in structure and/or stereochemistry.

### Expression of Cdk5 in endothelial cells, cytotoxicity and proliferation

**Figure 2:**
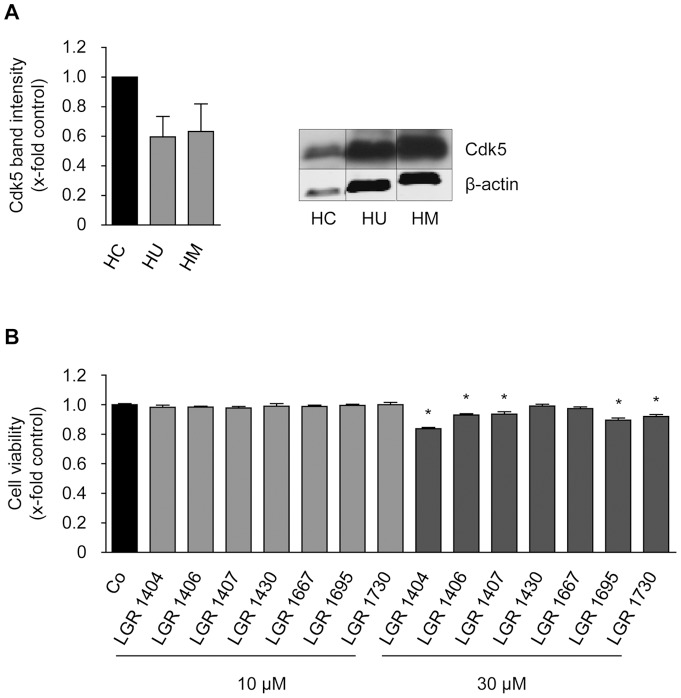
Cdk5 is expressed in HUVECs and HMEC-1 cells, and the compounds are not cytotoxic to endothelial cells at concentrations used in functional assays. A: Cdk5 protein expression in endothelial cells in comparison to human cortex. Cdk5 protein amount was analyzed by Western blot in samples of human cortex (HC), confluent HUVECs (HU) and HMEC-1 (HM). β-actin served as a loading control and for normalization of protein amount. Relative quantification (left panel) and one representative image (right panel) of three individual blots are shown. Note the much lower protein loading in the HC sample in the right panel. (n = 3, mean ± SEM, p>0.05, One Way ANOVA, Dunnett). B: Confluent HUVECs were treated for 16 h with 10 or 30 µM of the indicated compounds or left untreated as control. After addition of CellTiter-BlueTM Reagent, cells were incubated for 4 h and fluorescence was measured at 560 nm (n = 3, mean ± SEM, * p<0.05, One Way ANOVA, Dunnett).

**Figure 3:**
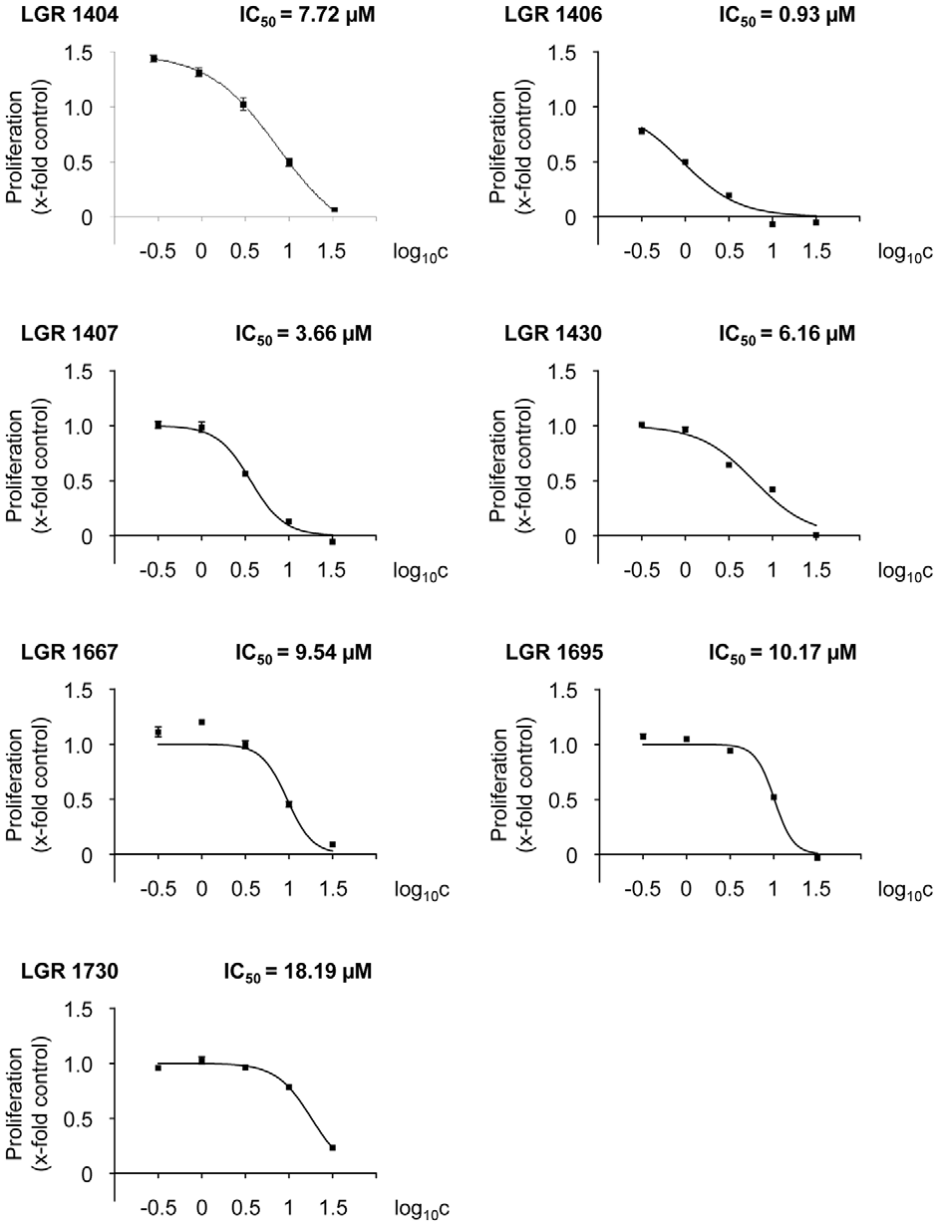
All tested compounds concentration-dependently inhibit proliferation of endothelial cells. HMEC-1 cells were seeded to a density of 1,500 cells/well. After 24 h of incubation, a zero point control was stained with crystal violet. The remaining cells were left untreated as positive control or treated with 0.3-1-3-10–30 µM of the indicated compounds. After 72 h additional incubation, relative proliferation was determined by crystal violet staining and quantified as absorption at 550 nm.

**Figure 4:**
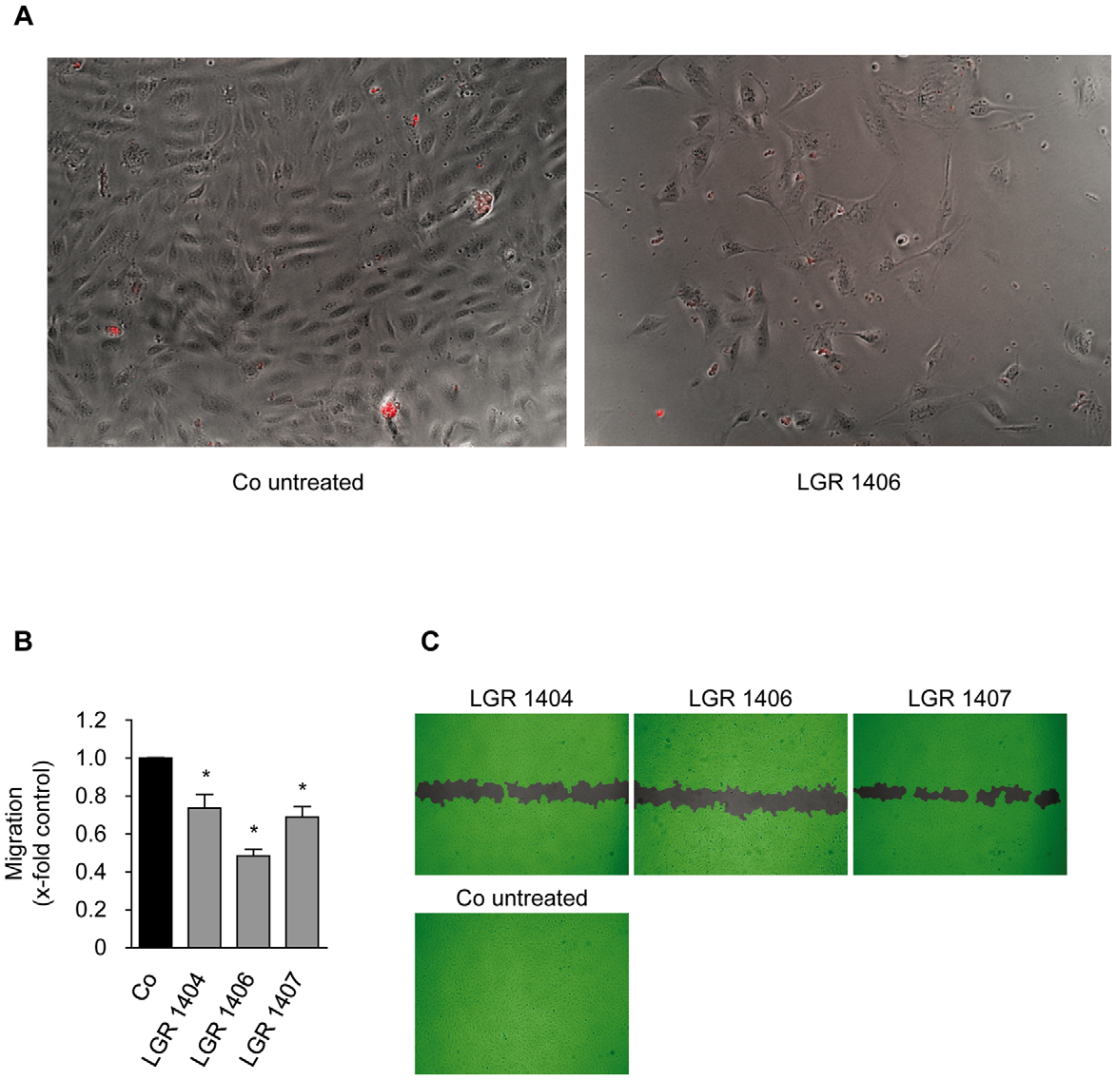
LGRs do not reduce cell numbers by inducing cell death and LGR 1404, 1406 and 1407 significantly reduce endothelial cell migration at 10 µM. A: HMEC-1 cells were seeded in the same density as for the proliferation assay and treated with the respective compound at 30 µM for 72 h to test whether reduced cell numbers in the proliferation assay are due to inhibition of proliferation or to excessive cell death. No increase of detached, obviously dead or propidium iodide positive cells was detected. B and C: Confluent layers of HUVECs were scratched and the cells were allowed to migrate for 16 h in the presence or absence of 10 µM of the compounds. B: The columns indicate the area re-covered by migrating cells (n = 3, mean ± SEM, * p<0.05, One Way ANOVA, Dunnett). C: Scratches at endpoint, representative images taken out of three experiments.

Since Cdk5 has not been described to be present in HMEC-1 cells before, we compared the expression of Cdk5 in HMEC-1 to that in HUVECs and in neuronal tissue (human cortex lysate as positive control). HMEC-1 and HUVC expressed Cdk5 to a similar amount, but to a lesser degree that cortex (about by half, Figure 2A). To detect potential toxic effects on non-proliferating endothelial cells in order to assess the systemic applicability of the compounds, the impact of the novel Cdk inhibitors on viability was tested in confluent monolayers. No reduced cell viability was found for 10 µM of each of the inhibitors in comparison to control. By contrast, 30 µM of LGR 1404, 1406, 1407, 1695 and 1730 displayed a weak but significant decrease of viability (Figure 2B). Therefore, in the functional assays, the effects at 10 µM were used as selection criterion. As a first screening step towards an anti-angiogenic potential, the novel inhibitors were then tested in crystal violet proliferation assays with the endothelial cell line HMEC-1. All seven compounds concentration-dependently reduced endothelial cell proliferation (Figure 3), with an IC_50_ between approximately 1 µM (LGR 1406) and 20 µM (LGR 1730). Microscopic analysis after 72 h of incubation with the compounds at a concentration of 30 µM at the same cell density as in the proliferation assay showed no increase of morphologically altered, dead or detached cells, and no loss of membrane integrity (Figure 4A with LGR 1406 as representative compound, other compounds not shown).

### Cell migration

**Figure 5:**
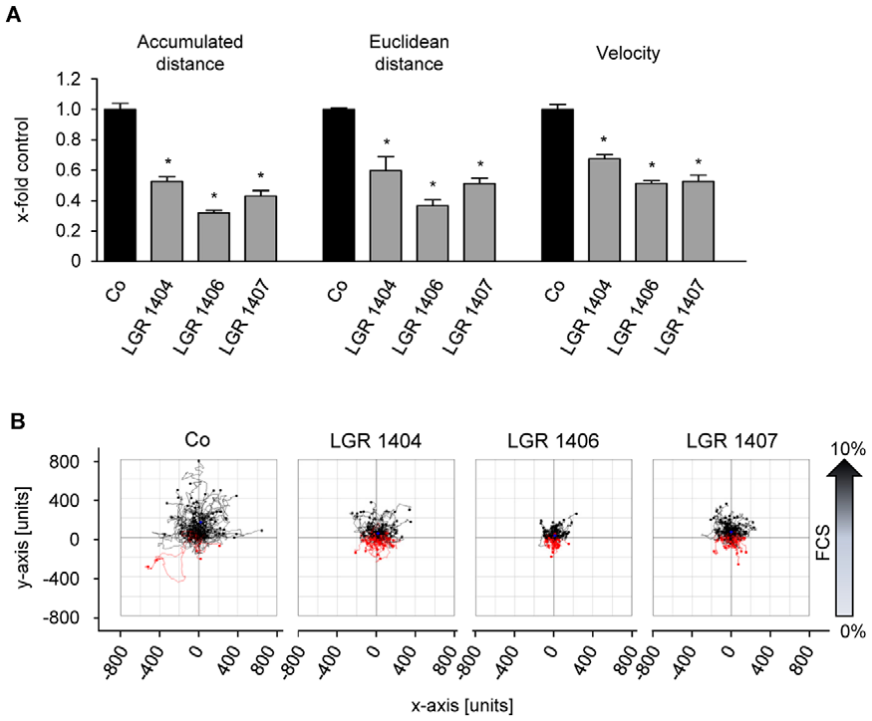
LGR 1404, 1406 and 1407 decrease chemotaxis of endothelial cells. Chemotaxis of HUVECs in the presence or absence of 10 µM of the indicated compounds was determined in µ-slides Chemotaxis. A: Quantitative evaluation of accumulated and euclidean distance, velocity and y-forward migration index (n = 3, mean ± SEM, * p<0.05, One Way ANOVA, Dunnett). B: Representative cell tracking plots of untreated and treated cells.

Endothelial cell migration is the subsequent crucial step in angiogenesis after the activation of the quiescent endothelial cells to proliferate. All seven LGR compounds were tested in the scratch assay for their effect on migration at the sub-toxic concentration of 10 µM. Only LGR 1404, 1406 and 1407 were able to significantly decrease endothelial cell migration at this concentration. Treatment with 10 µM of the most potent substances, LGR 1406 and 1407, reduced migration by 51% and 31%, respectively (Figure 4B and C). In order to obtain more detailed information on their mode of action on migration, the active compounds were also tested in a chemotactic gradient of FCS. Accumulative (as marker for overall cell migration) and euclidean migration distance (as indicator of directionality), as well as cell velocity were analyzed. All three compounds led to a decrease in all determined migration indices (Figure 5).

### Tube formation

**Figure 6:**
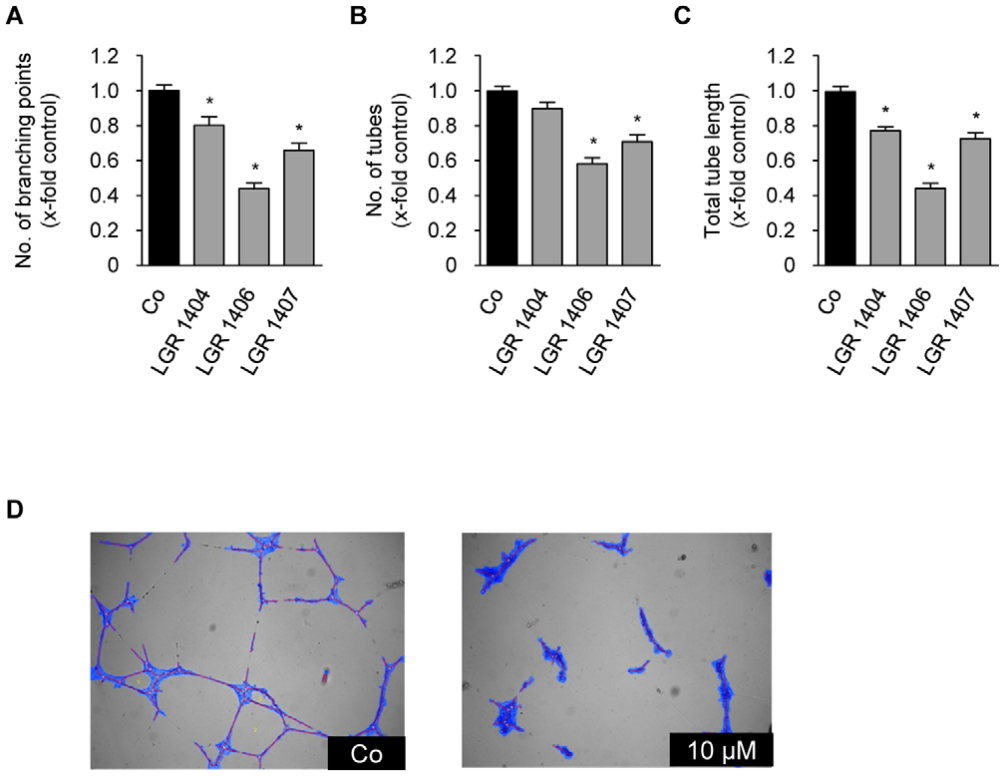
LGR 1404, 1406 and 1407 inhibit tube formation at 10 µM. HUVECs were seeded onto a matrix of growth-factor reduced Matrigel^TM^ in the presence or absence of the respective concentration of the compounds. After 16 h of incubation, images were taken and tube characteristics were quantified. A: Number of tubes (n = 3, mean ± SEM, * p<0.05, One Way ANOVA, Dunnett). B: Number of branching points (n = 3, mean ± SEM, * p<0.05, One Way ANOVA, Dunnett). C: Tube total length (n = 3, mean ± SEM, * p<0.05, One Way ANOVA, Dunnett). D: Representative images of the tube formation assay and the software based tube recognition (structures identified as tubes are blue) in a control (Co, left panel) and after treatment with LGR 1406 (10 µM, right panel).

The most powerful compounds from the migration experiments, LGR 1404, 1406 and 1407 were chosen for tube formation assays. 10 µM of LGR 1404, 1406 and 1407 all showed a significant reduction of tube and branching point numbers as well as of total tube length (Figure 6). LGR 1406 and 1407 again showed the strongest effects. 10 µM of LGR 1406 decreased tube length and number of branching points by 56%, and the tube number by 42%. Treatment with 10 µM of LGR 1407 resulted in an about 30% reduction of tube number and total tube length; and to a 35% reduction in the number of branching points (Figure 6).

### CAM Assay

**Figure 7:**
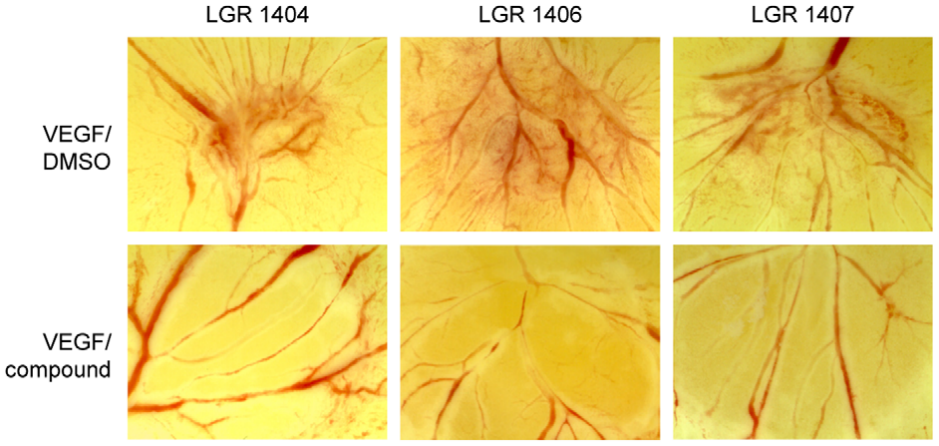
LGR 1404, 1406 and 1407 completely inhibit VEGF-induced vessel formation in the chorioallantoic membrane (CAM) assay. Fertilized white leghorn eggs were incubated for 72 h at 37°C in humidified atmosphere. After transferring the growing embryo into Petri dishes, a second incubation period of 72 h followed. At day 6, cellulose discs with 2.5 ng VEGF/250 nmol compound were placed on the membrane. 2.5 ng VEGF/DMSO was used as control. Pictures were taken after 24 h of stimulation. Representative images out of at least three experiments are shown.

The anti-angiogenic potency of the three most effective compounds has been evaluated *in vitro* so far. In order to substantiate these findings *in vivo*, chorioallantoic membrane (CAM) assays were performed with LGR 1404, 1406 and 1407. All three compounds completely abolished VEGF induced vessel formation (Figure 7).

### *In vitro* kinase profile

We found that LGR 1404, 1406 and 1407 were the most potent compounds in all angiogenesis assays. Therefore, it was of interest to see which kinases, especially which Cdks, are inhibited by those compounds. The kinase profiling was performed by ProQinase (Freiburg, Germany) for LGR 1406 and 1407. For LGR 1404 kinase profiling has recently been published previously [23]. LGR 1406 and 1407 were tested for their IC_50_ in a panel of 24 kinases, including the Cdk1, Cdk2, Cdk4, Cdk5, Cdk6, Cdk7 and Cdk9. The other kinases tested were PTK6, EGFR, FAK, FGFR1 and FGFR2, NLK, PAK4, VEGFR1 and VEGFR2, MEK1, ROCK1, RAF1, ALK, RSK3, AURKA, and AMPKα1.

The IC_50_ [M] of LGR 1406 and LGR 1407 for the Cdk/Cyclin complexes are shown in Table 1. Both compounds inhibit mainly Cdk2 and Cdk5, and to some extent Cdk9 and Cdk1. Concerning the other tested kinases, FAK, PAK4, RSK3 and Aurora kinase A are inhibited by LGR 1406 with an IC_50_ below 1×10^−5^ M. LGR 1407 only inhibits Aurora kinase A (IC_50_ ≤1×10^−5^ M) in addition to the Cdks displayed in Table 1.

**Table 1:** Cdk inhibition profile of LGR 1406 and 1407.

	Cdk1/CycB	Cdk2/CycA	Cdk2/CycE	Cdk4/CycD1	Cdk5/p25	Cdk6/CycD1	Cdk7/CycH/MAT1	Cdk9/CycT
**LGR 1406**	3.2×10^−6^	9.9×10^−7^	5.9×10^−7^	1.5×10^−5^	4.4×10^−7^	>10^−4^	>10^−4^	10^−6^
**LGR 1407**	5.8×10^−6^	1.5×10^−6^	9.9×10^−7^	6.6×10^−5^	1.6×10^−6^	9.1×10^−5^	>10^−4^	1.9×10^−6^

The numbers given are IC_50_ values (molar). Both compounds show an increased selectivity for Cdk2 and Cdk5.

### Formation of lamellipodia and transcolation of Rac1 to lamellipodia

**Figure 8:**
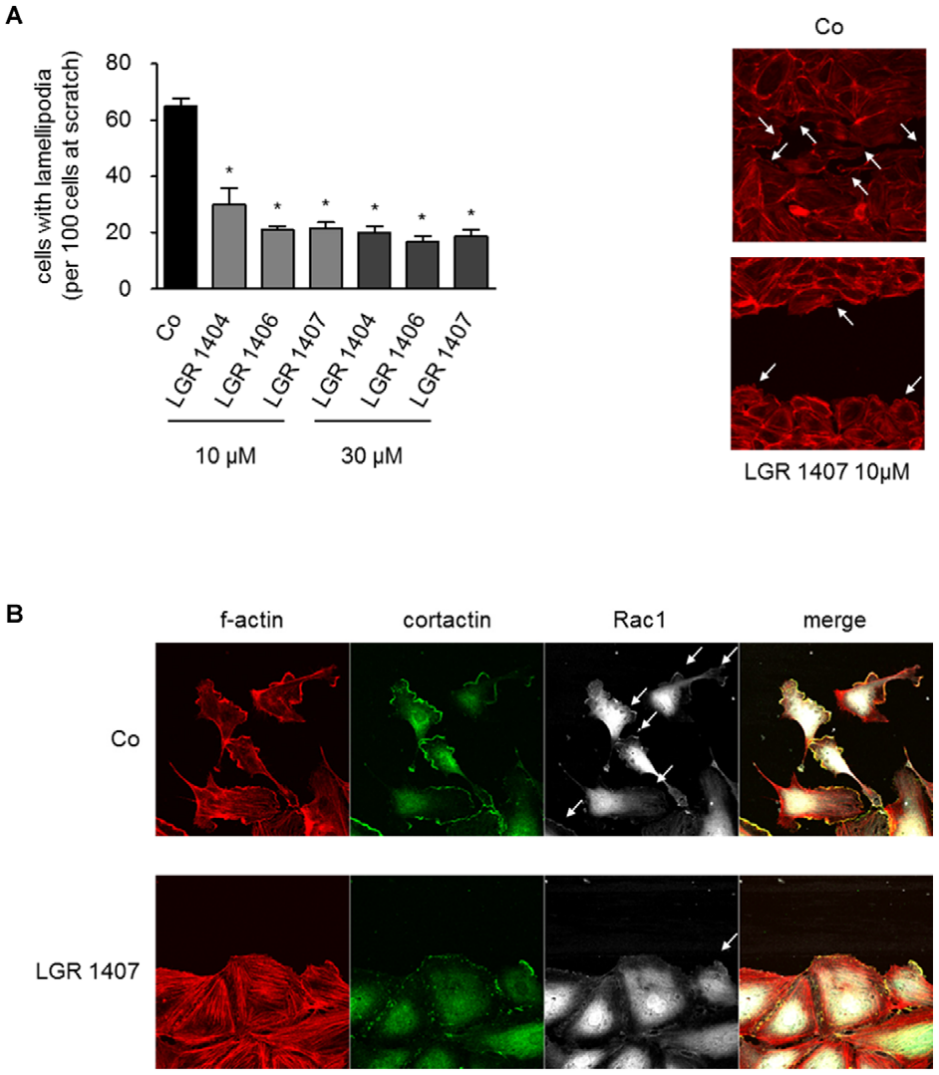
LGR 1404, 1406 and 1407 decrease lamellipodia formation in migrating cells, and inhibit recruitment of Rac1 to lamellipodia. A: Confluent layers of HUVECs were scratched and the cells were allowed to migrate for 8 h in the presence or absence of the respective concentration of the compounds, until clear lamellipodia formation was visible in the control. The actin cytoskeleton was then stained with rhodamin-phalloidine and fluorescence images (10x magnification) of the scratches were taken. Left panel: For quantitative evaluation of lamellipodia formation, cells with prominent lamellipodia and ruffles were counted in relation to the number of cells at the scratch front (n = 3, mean ± SEM, * p<0.05, One Way ANOVA, Dunnett). Right panel: Representative images of the scratch front (f-actin, 40x magnification). B: Cells treated like in A were fixed and stained for f-actin (red), cortactin (green) and Rac1 (white). Representative images out of three experiments are shown.

In order to get an insight into the mechanism underlying the anti-angiogenic action of the three most potent LGR, we analyzed their effect on the formation of lamellipodia in migrating endothelial cells. LGR 1404, 1406 and 1407 significantly diminished the formation of lamellipodia by 54% (LGR 1404) to 67% (LGR 1406 and 1407) at 10 µM. This can be seen in the respective images stained for f-actin (Figure 8A). To substantiate this finding, we examined the localization of Rac1 to the cell front of migrating cells. In immunofluorescence stainings we found a decreased Rac1 localization to lamellipodia as displayed in Figure 8B. Cortactin served as a marker protein for lamellipodia.

## Discussion

We tested seven derivatives of the classical Cdk inhibitor roscovitine as anti-angiogenic compounds in an approach where the effect on endothelial migration was the crucial selection criterion. This setting was chosen, since we have previously shown that roscovitine and derivatives thereof had an anti-angiogenic potential, which was based on the reduction of endothelial cell motility *via* inhibition of Cdk5 [15], [24]. The three compounds which performed best in these and other functional assays (tube formation and directed migration in a chemotactic gradient) in the present work, LGR 1404, 1406 and 1407, also proved their anti-angiogenic potency *in vivo* in CAM-assays, where they completely inhibited VEGF-induced vessel formation. Thus, we have identified three potent novel roscovitine derivatives that display increased anti-angiogenic activity in comparison to their mother substance roscovitine: while roscovitine itself only started to reduce proliferation at a concentration of 30 µM [24], the three compounds LGR 1404, 1406 and 1407 had IC50 values of 7.72, 0.93 and 3.66 µM, respectively. Concerning migration, 10 µM roscovitine yielded only 20% reduction [15], while the compounds in the present work showed an inhibition between 30 and 50% at an equimolar concentration. A similar difference was observed during tube formation [15]. Roscovitine itself is termed a “pan selective” inhibitor of Cdks, since it mainly addresses Cdk1, Cdk2, Cdk5, Cdk7 and Cdk9. The selectivity data depend on the kinase panel referred to [14], [23], [25], [26]. LGR 1407 is equally potent in inhibition of Cdk2 and Cdk5, and inhibits Cdk1 and Cdk9 to some extent. LGR 1406 is by one order of magnitude more selective towards Cdk5 and Cdk2 in comparison to Cdk1 and Cdk9. Both compounds inhibited preferably Cdks in our kinase panel, with LGR 1407 showing a higher Cdk selectivity. Comparing the two most powerful compounds LGR 1406 and 1407, the lower IC_50_ for Cdk5 and the higher selectivity for Cdk5 (and Cdk2) of LGR 1406 mirror the effect in the angiogenesis assays. LGR 1407 is more selective towards Cdk5 in comparison to LGR 1404, which mainly inhibits Cdk2 (IC_50_ for Cdk2 0.22 µM, for Cdk5 0.94 µM according to Jorda *et al*. [23]). This is probably the reason why LGR 1404 is the least potent anti-angiogenic compound of the three with regard to the *in vitro* data.

Since we have previously shown by silencing experiments that Cdk5 influences endothelial migration *via* a reduction of activated Rac1 [15], a small GTPase of central importance for lamellipodia formation and cell motility, we also determined the effect of LGR 1404, 1406 and 1407 on lamellipodia formation and Rac1 localization, as an indicator of Cdk5 inhibition. Due to their respective effects, we suggest that their mode of action is indeed the potent inhibition of Cdk5 and not Cdk2. The lower selectivity of LGR 1404 for Cdk5 becomes also apparent in the lamellipodia quantification and the Rac1/lamellipodia immunofluorescence images: the disruption of lamellipodia and the effect on Rac1 is not that prominent as with LGR 1406 and LGR 1407.

In order to adapt the structure of the Cdk inhibitors for optimal anti-angiogenic potential, the relation of structural changes and anti-angiogenic effect is of interest. For the LGR compounds as roscovitine derivatives, the structure was modified in three points:

1. Changing the purine scaffold to a pyrazolo[4,3-*d*]pyrimidine: In general, the change of the scaffold led to a higher anti-angiogenic potency of the substances. All substances chosen for further evaluation after the migration assay share the pyrazolo[4,3-*d*]pyrimidine scaffold. Direct comparison of the potency of roscovitine and its pyrazolo[4,3-*d*]pyrimidine bioisoster, LGR 1404, substantiates this observation. The only compound tested with a purine scaffold, LGR 1730, showed the weakest effect on proliferation and no significant impact on migration (data not shown).
2. Ortho-amino function in the aminobenzyl group at C6 (purine) or C7 (pyrazolo[4,3-*d*]pyrimidine): The presence of an amino group rather seems to decrease the anti-angiogenic potential of the compounds. The compounds LGR 1430 and LGR 1492 differ from LGR 1406 and LGR 1404, respectively, only in the presence of the amino function, and show both weaker effects. This is especially obvious in the comparison of LGR 1406 and LGR 1430 as LGR 1406 was the most potent compound in the assays, whereas LGR 1430 showed no detectable effect on migration at 30 µM (data not shown).
3. Variation of the side chain at C2 (purine) or C5 (pyrazolo[4,3-*d*]pyrimidine): Evaluating the impact of different side chains on the anti-angiogenic effect is difficult as the compounds differ from roscovitine in more than one structural property and no direct comparison is possible. By trend, a bulky side chain like the substituted sec-butyl- (e.g. LGR 1404) or cyclohexyl- (e.g. LGR 1406) groups seem to increase anti-angiogenic potency.

In conclusion, we have demonstrated that LGR 1404, 1406 and 1407 are able to potently inhibit angiogenesis *in vitro* via a Cdk5-dependent mechanism and show a higher potency and selectivity for Cdk5 in comparison to the established Cdk5 inhibitor roscovitine.

Their impact on Cdk5 parallels the efficacy in the angiogenesis assays which supports the strategy of Cdk5 inhibition as a powerful new approach in anti-angiogenic therapy. For the further development of anti-angiogenic roscovitine derivatives, comparison of the structures of the tested LGR inhibitors shows a positive correlation to anti-angiogenic potency for the pyrazolo[4,3-*d*]pyrimidine scaffold and a negative correlation for an additional amino function in the benzyl group.

## Materials and Methods

### Studied compounds and general chemical procedures

7-Benzylamino-5(*R*)-[(1-hydroxymethyl)propylamino]-3-isopropyl-1(2)*H*-pyrazolo[4,3-*d*]pyrimidine (compound **LGR1404**), 7-benzylamino-3-isopropyl-5-methylsulfonyl-1(2)*H*-pyrazolo[4,3-*d*]pyrimidine and 7-(2-aminobenzylamino)-3-isopropyl-5-methylsulfonyl-1(2)H-pyrazolo-[4,3-*d*]-pyrimidine (precursors of the synthesis described here) have been prepared according to the published procedures [23]. All other studied compounds have been synthesized according to the scheme shown in Figure 1B.

Melting points were determined on a Kofler block and are uncorrected. NMR spectra have been measured on a Bruker 300 MHz spectrometer (75 MHz, respectively for ^13^C) in DMSO-*d_6_* or CDCl_3_ at 303 K. The residual solvent signal has been used as an internal standard (δ_H_ 2.500, δ_C_ 39.60 for DMSO- *d_6_*, or δ_H_ 7.265, δ_C_ 77.00 for CDCl_3_). ^1^H NMR, ^13^C NMR, COSY, HSQC, HMBC have been measured using standard manufacturers' software (Varian Inc., Palo Alto, USA). Chemical shifts are given in δ-scale [ppm] and coupling constants in Hz. Some ^13^C signals of the heterocycle were not visible due to bad relaxation. ESI or APCI mass spectra were determined using a Waters Micromass ZMD mass spectrometer (direct inlet, coin voltage 20 V). Merck silica gel Kieselgel 60 (230–400 mesh) was used for column chromatography. Compound purity was determined by elemental analyses (0.4%) or HPLC-MS analysis and was confirmed to be >95% for all compounds.

### 7-Benzylamino-5(*R*)-[(2-hydroxypropyl)amino]-3-isopropyl-1(2)*H*-pyrazolo[4,3-d]pyrimidine (compound LGR1667)

Methylsulfone **1** (0.25 g, 0.72 mmol) and *R*-(-)-3-amino-2-propanole 0.6 mL (7 mmol) were heated in sealed ampoule for 5 h to 120°C. Excess of the amine was evaporated at a temperature below 70°C and the residue was partitioned between CHCl_3_/H_2_O. The combined organic phases were dried with magnesium sulfate and evaporated. Product was purified by column flash chromatography on silica gel stepwise with 4, 5, 6, 7, 8% MeOH in CHCl_3_. The product was obtained in a non-crystallisable amorphous and colourless glass 72 mg, yield 29%, [α]_D_  = −24.2° (c = 1 g/l, CHCl_3_, 20°C) MS ESI^+^: [M+H]^+^  = 341,4 (100%), [2M+H]^+^  = 681 (4%), MS ESI^−^: [M−H]^−^  = 339,3 (100%). ^1^H-NMR (300 MHz, CDCl_3_): 1.182 d (3H, *J* = 6.2 Hz, 23), 1.202 d (6H, *J* = 6.6 Hz, 19), 3.114 sept (1H, *J* = 7.0 Hz, 18), 3.260–3.410 m (2H, 21), 3.974 m (1H, 22), 4.666 s (2H, 11), 5.400 bs (1H, NH or OH), 7.200–7.276 m (5H, ArH). ^13^C-NMR (75 MHz, CDCl_3_): 20.2 (23), 20.9 (11), 25.4 (18), 43.7 (11), 49.9 (21), 69.2 (22), 126.5, 126.8, 127.2, 128.0, 137.5, 158.4. Anal. (C_18_H_24_N_6_O) C, H, N.

### 5-[(2-*E/Z*-aminocyclohexyl)amino]-7-benzylamino-3-isopropyl-1(2)*H*-pyrazolo[4,3-d]pyrimidine (compound LGR1406)

Methylsulfone **1** (0.3 g, 0.87 mmol) and (E/Z)-1,2-diaminocyclohexane 2.8 mL were heated in sealed ampoule for 8 h to 145°C. Excess of the amine was evaporated at a temperature below 70°C and the residue was partitioned between CHCl_3_/H_2_O. The combined organic phases were dried with sodium sulfate and evaporated. Product was purified by column flash chromatography on silica gel stepwise with 8, 10, 12, 14% MeOH in CHCl_3_ with a trace of concentrated NH_4_OH. The product was obtained in a non-crystallisable amorphous colorless glass 49 mg, yield 15%, MS ESI^+^: [M+H]^+^  = 380.4 (100%), MS ESI^−^: [M−H]^−^  = 378.4 (100%). ^1^H-NMR (300 MHz, DMSO-d*6*): 1.01–1.31 m (3H, 24a, 24b, 25a), 1.32 d (6H, *J* = 7.0 Hz, 19), 1.44–1.68 m (3H, 23a, 23b, 26a), 1.84 bd (1H, 25b), 2.01 bd (1H, 26b), 2.54 m (1H, 22), 3.16 sept (1H, *J* = 7.0 Hz, 18), 3.43 m (1H, 21), 4.68 s (2H, 11), 5.82 d (1H, *J* = 7.7 Hz, 20), 7.25 t (1H, 15), 7.34 t (2H, 14+16), 7.39 d (2H, 13+17), 7.75 bs (1H, 10), 11.98 bs (1H, 1). ^13^C-NMR (75 MHz, DMSO-d*6*): 21.6 (19); 21.7 (19); 24.8 (24); 24.9 (25); 25.8 (18); 31.9 (26); 34.3 (23); 43.0 (11); 54.0 (22); 57.1 (21); 126.8 (15); 127.5 (13+17); 128.3 (14+16); 136.0; 139.6 (12); 145.7; 150.6 (7); 157.4; 158.1; 168.6. Anal. (C_21_H_29_N_7_) C, H, N.

### 5-[(4-*E*-aminocyclohexyl)amino]-7-benzylamino-3-isopropyl-1(2)*H*-pyrazolo[4,3-d]pyrimidine (compound LGR1407)

Methylsulfone **1** (0.3 g, 0.87 mmol) and (*Z*)-1,4-diaminocyclohexane 4.5 g were heated in sealed ampoule for 8 h to 145°C. Excess of the amine was evaporated at a temperature below 70°C and the residue was partitioned between CHCl_3_/H_2_O. The combined organic phases were dried with sodium sulfate and evaporated. Product was purified by column flash chromatography on silica gel stepwise with 8, 10, 12, 14% MeOH in CHCl_3_ with a trace of concentrated NH_4_OH. Product was obtained in a form of non-crystallisable amorphous colourless glass 57 mg, yield 17%, MS ESI^+^: [M+H]^+^  = 380.3 (100%), MS ESI^−^: [M−H]^−^  = 378.4 (100%). ^1^H-NMR (300 MHz, DMSO-d*6*): 1.04–1.27 m (4H, 22, 23), 1.32 d (6H, *J* = 7.0 Hz, 19), 1.76 bd (2H, *J* = 10.0 Hz, 25), 1.90 bd (2H, *J* = 10.0 Hz, 26), 2.54 m (1H, 24), 3.15 sept (1H, *J* = 7.0 Hz, 18), 3.6 m (1H, 21), 4.66 d (2H, *J* = 5.3 Hz, 11), 5.71 d (1H, *J* = 7.9 Hz, 20), 7.24 t (1H, 15), 7.33 t (2H, 14+16), 7.38 d (2H, 13+17), 7.73 t (1H, *J* = 5.3 Hz, 10), 12.13 bs (1H, 1). ^13^C-NMR (75 MHz, DMSO-d*6*): 21.6 (19); 25.8 (18); 31.3 (22+21); 34.8 (23+25); 42.9 (11); 49.4 (21); 49.9 (24); 121.0; 126.8 (15); 127.4 (13+17); 128.2 (14+16); 139.6 (12); 140.0 (3); 144.7 (9); 150.6 (7); 157.3 (5). Anal.(C_21_H_29_N_7_) C, H, N.

### 7-(2-Aminobenzyl)amino-5(*R*)-[(2-hydroxypropyl)amino]-3-isopropyl-1(2)*H*-pyrazolo[4,3-d]pyrimidine (compound LGR1730)

Methylsulfone **2** (0.4 g, 1.1 mmol) and R(-)-3-amino-2-propanole 1 mL were heated in a sealed ampoule for 5 h to 120°C. Excess of the amine was evaporated at a temperature below 70°C and the residue was partitioned between CHCl_3_/H_2_O. The combined organic phases were dried with magnesium sulfate and evaporated. Product was purified by column flash chromatography on silica gel stepwise with 4, 5, 7, 10% MeOH in CHCl_3_, crystallized from CHCl_3_-Et_2_O, mp 186–189°C, 71 mg, yield 18%, [α]_D_  = −14.8° (c = 1 g/l, CHCl_3_, 20°C), MS ESI^+^: [M+H]^+^  = 356.3 MS ESI^−^: [M−H]^−^  = 354.3 (100). ^1^H-NMR (300 MHz, CDCl_3_): 1.186–1.311 m (9H, 19+23), 3.185 sept (1H, *J* = 7.0 Hz, 18), 3.370–3.530 m (2H, 21), 3.920–4.061 m (1H, 22), 4.657 s (2H, 11), 6.150 bs (1H, NH), 6.622–6.699 m (2H, ArH), 7.058–7.141 m (2H, ArH), 8.199 s (1H, OH). ^13^C-NMR (300 MHz, CDCl_3_): 19.9 (23), 20.3 (19), 25.2 (18), 44.9 (11), 49.8 (21), 66.4 (22), 115.4, 117.7, 121.2, 126.5, 128.7, 130.2, 133.9, 144.7, 153.2, 161.3, 175.0. Anal. (C_18_H_25_N_7_O) C, H, N.

### 7-(2-Aminobenzyl)amino-5(*S*)-[(2-hydroxypropyl)amino]-3-isopropyl-1(2)*H*–pyrazolo[4,3-d]pyrimidine (compound LGR1695)

The compound has been prepared using the same synthetic process as for R antipode. Analytic data are the same as for R antipode, with the exception of [α]_D_  = +15.2° (c = 1 g/l, CHCl_3_).

### 7-(2-Aminobenzyl)amino-5-[(2-*E/Z*-aminocyclohexyl)amino]-3-isopropyl-1(2)*H*-pyrazolo-[4,3-d]pyrimidine (compound LGR1430)

Methylsulfone **2** (0.4 g, 1.1 mmol) and (*E/Z*)-1,2-diaminocyclohexane 5 mL were heated in a sealed ampoule for 8 h to 145°C. Excess of the amine was evaporated at a temperature below 70°C and the residue was partitioned between CHCl_3_/H_2_O. The combined organic phases were dried with sodium sulfate and evaporated. Product was purified by column flash chromatography on silica gel stepwise with 8, 10, 12, 14% MeOH in CHCl_3_ with a trace of concentrated NH_4_OH. Product was obtained in a form of non-crystallisable amorphous colourless glass 55 mg, yield 13%, MS ESI^+^: [M+H]^+^  = 395.3 MS ESI^−^: [M−H]^−^  = 393.4 (100). ^1^H-NMR (300 MHz, DMSO-d6): 1.03–1.26 m (3H, 24a, 24b, 25a), 1.32 d (6H, *J* = 7.0 Hz, 19), 1.51–1.68 m (3H, 23a, 23b, 26a), 1.84 d (1H, *J* = 10.0 Hz, 25b), 2.06 d (1H, *J* = 12.4 Hz, 26b), 2.54 m (1H, 22), 3.15 sept (1H, *J* = 7.0 Hz, 18), 3.29 bs (2H, 27), 3.38 m (1H, 21), 4.53 d (2H, 11, *J* = 5.3 Hz), 5.14 bs (2H, 28), 5.86 d (1H, *J* = 7.7 Hz, 20), 6.51 t (1H, *J* = 7.3 Hz, 16), 6.64 d (1H, *J* = 7.9 Hz, 14), 6.97 t (1H, *J* = 7.9 Hz, 15), 7.12 d (1H, *J* = 7.3 Hz, 17), 7.50 bs (1H, 10), 11.98 bs (1H, 1). ^13^C-NMR (75 MHz, DMSO-d6): 21.5 (19); 21.6 (19); 24.9 (24); 25.0 (25); 25.8 (18); 31.9 (26); 34.7 (23); 39.7 (11); 54.1 (22); 57.4 (21); 114.6 (14); 115.7 (16); 121.9 (12); 123.1; 127.3; 127.9 (15); 129.5 (17); 139.8; 145.7 (13); 150.4; 158.1; 160.7. Anal (C_21_H_30_N_8_) C, H, N.

### Cell culture

Endothelial cells (HMEC-1 Human microvascular endothelial cells and HUVECs Human umbilical vein endothelial cells) were cultured in growth medium (ECGM, Promocell, Heidelberg, Germany) containing 10% fetal calf serum as previously described [27]. The cell line HMEC-1 (CDC/EU.HMEC-1) was kindly provided by the Centers for Disease Control and Prevention (Atlanta, GA, USA) [28]. HUVECs were freshly isolated from umbilical cords placed in PBS containing Ca^2+^/Mg^2+^, penicillin (100 U/ml), and streptomycin (100 µg/ml). The umbilical vein was cannulated, flushed with PBS+Ca^2+^/Mg^2+^, filled with 0.1 g/l collagenase A, and incubated for 45 min at 37°C. The eluted cell suspension was centrifuged (1,000 rpm, 5 min). Afterwards, cells were resuspended and plated in a 25 cm^2^ flask (passage #0). After reaching confluency, cells were trypsinized and plated in a 75 cm^2^ flask. Unless otherwise indicated, experiments were performed using cells at passage #3. All patients with no special risks before giving birth were routinely asked for their consent for using anonymous tissue materials for non-profit research. Since no risk or additional encumbrance was induced by collecting the umbilical cords, the local ethics committee had not to be informed in this case.

### Western blot

Samples were lysed in RIPA buffer, frozen at −80°C, centrifuged at 14,000 rpm for 10 min at 4°C, and heated in Laemmli buffer at 95°C for 5 min. Equal amounts of protein were loaded on discontinuous polyacrylamide gels, consisting of a separation and a stacking gel, and separated using the Mini-PROTEAN 3 electrophoresis module (Bio-Rad, Munich, Germany). The molecular weight of proteins was determined by comparison with a prestained protein ladder (PageRulerTM, Fermentas, St. Leon-Rot, Germany). After protein separation, proteins were transferred to a nitrocellulose membrane (Hybond-ECLTM, Amersham Bioscience, Freiburg, Germany) by tank electroblotting in the Mini Trans-Blot® system (Bio-Rad, Munich, Germany). Cdk5 was detected by incubation with a polyclonal rabbit antibody (C-8) from Santa Cruz. Equal loading was controlled by detecting β actin mouse polyclonal C4 from Millipore).

### Cell viability assay

HUVECs were seeded in 96-well plates. After reaching confluency, the cells were treated for 16 h with the indicated compounds or left untreated as control. After addition of CellTiter-Blue^TM^ Reagent (Promega Corporation, Madison, WI, USA), cells were incubated for additional 4 h and fluorescence was measured at 560 nm in a plate-reading photometer (SpectraFluor Plus; Tecan, Crailsheim, Germany).

### Proliferation assay

HMEC-1 were seeded into 96-well-plates (1,500 cells/well). After 24 h, the cells were stimulated with the indicated compounds. At the same time point, cells in a reference plate were stained with crystal violet, serving as initial cell number. After 72 hours of stimulation, cells were fixed and stained with crystal-violet solution for 10 minutes, washed with water, and air dried. Crystal violet was eluted with dissolving buffer and absorbance was measured at 550 nm (Tecan Sunrise Absorbance reader, TECAN, Crailsheim, Germany). In a parallel series of experiments, cells seeded at the same cell density as in the proliferation assays were treated with 30 µM of the respective compound for 72 h. Afterwards, 10 µg/ml propidium iodide was added for 30 min to counterstain cells for membrane integrity. Phase contrast and fluorescence images were obtained with an inverted Nikon microscope (Ti, Nikon, Düsseldorf, Germany) equipped with a 10x objective.

### Scratch assay (migration assay)

HUVECs were seeded into a 24-well plate. After reaching confluency, cells were scratched using a 100 µl pipette tip. The wounded monolayers were washed twice with PBS containing Ca^2+^/Mg^2+^; then growth medium containing the indicated concentration of the compounds was added. After 16 h of migration, cells were washed and subsequently fixed with 4% paraformaldehyde. In previous experiments we have shown that under these conditions cell proliferation does not contribute substantially to scratch closure [15]. Images were taken using the TILLvisON system (TILL Photonics GmbH, Gräfelfing, Germany) and a CCD-camera connected to an Axiovert 200 microscope (Zeiss, Oberkochen, Germany). Quantitative image analysis of the cell covered area was done by Wimasis GmbH, Munich. A completely closed scratch was set as 100% migration, the area of the original open scratch was set as 0% migration.

### Chemotaxis assay

The effect of the LGR compounds on endothelial cell chemotaxis was determined using Collagen IV coated μ-slides Chemotaxis (ibidi, Martinsried, Germany). HUVECs (5×10^6^ cells per ml) were seeded into the observation channel of the slides according to the manual of the manufacturer, and the cells were allowed to attach for 4 hours. Then, the chambers of the chemotaxis slide were completely filled with serum-free medium M 199; and growth medium containing 30% FCS was added to one chamber in order to generate an FCS-gradient from 0% to 10%. 10 µM of the indicated compounds were added both to the M199 and to the 30% FCS. Chemotaxis was observed over 20 hours by live-cell imaging with a Zeiss LSM 510 META confocal microscope equipped with an incubator stage (EMBLem, Heidelberg, Germany). A time series was collected taking 1 picture every 10 minutes. For cell tracking and data analysis, the manual tracking plug-in (Fabrice Cordelieres) and the Chemotaxis and Migration Tool (Version 1.01, ibidi, Martinsried, Germany) for ImageJ were used. Euclidean distance (direct distance from the starting point to the end point) was used as an indicator of directionality of movement, accumulative distance (sum of all migrated distances from track to track) and velocity (mean velocity of every cell calculated as ratio between track to track distance and time) were used as a measure for total motility.

### Tube formation assay

Pre-cooled BD Matrigel^TM^ Matrix Growth Factor Reduced (GFR) (BD Biosciences, Heidelberg, Germany) was filled into the lower compartment of μ-slide Angiogenesis wells (ibidi GmbH, Martinsried, Germany) on ice. For polymerization of the Matrigel^TM^ Matrix, the slides were incubated at 37°C for 30 min. 12,000 HUVECs/well were seeded onto the Matrigel^TM^ and stimulated for 16 h. The extent of tube formation was determined by light microscopy using the TILLvisON system. Quantitative image analysis of tube length, number of branching points and tube number was performed with a software tool from Wimasis GmbH, Munich.

### Chorioallantoic membrane (CAM) assay

Fertilized white leghorn eggs were incubated for 72 h at 37°C in humidified atmosphere. After transferring the growing embryo into a Petri dish, a second incubation period of 72 h followed. At day 6, two cellulose discs, one with 2.5 ng VEGF (VEGF 165, human recombinant from PeproTech GmbH, Hamburg, Germany)/250 nmol compound and the other with 2.5 ng VEGF/DMSO as control were placed on one CAM. After 24 h of stimulation, the vascular structure in the stimulated areas of the CAM was visualized using a stereomicroscope and a CCD camera (Olympus, Munich, Germany) and pictures were taken. The cellulose disks were produced as follows: after preparing a cellulose solution (2.5% hydroxyethyl cellulose, 2% polyvinylpyrrolidone (PVP 17), 2% polyethylene glycol (PEG 400) in water), the mixture was autoclaved, resulting in a homogenized, clear solution. For each disk, 200 μl of the warm solution were filled into the preformed circles of the lid of a 96 well plate and allowed to polymerize under a laminar air flow for 48 h. Finally, the cellulose disks were removed using tweezers and stored in a sterile Petri dish until use.

### Quantification of lamellipodia and immunocytochemistry

Confluent layers of HUVECs were scratched and stimulated in 8-well μ-slides as described above. The cells were allowed to migrate for 8 h in the presence or absence of the respective concentration of the compounds, until clear lamellipodia formation was visible in the control. The actin cytoskeleton was then stained with rhodamine-phalloidin (Life Technologies, Darmstadt, Germany) and fluorescence images of the scratches were taken in 10x magnification with the Zeiss LSM 510 META. For quantitative evaluation of lamellipodia formation, cells with prominent lamellipodia and ruffles were counted with ImageJ (Cell Counter plug-in by Kurt De Vos) in relation to the total number of cells at the scratch front. The ratio was calculated as number of lamellipodia-positive cells per 100 cells at scratch front. In the same setting immunofluorescence staining was performed. Fixed cells were permeabilized for 2 min with 0.1% Triton X-100 in PBS. Cells were washed and unspecific binding was blocked with 0.2% BSA in PBS for 15 min. Thereafter, cells were incubated with primary antibody (cortactin: rabbit polyclonal, 1∶100, Cell Signaling, and Rac1: mouse monoclonal, 1∶100, Upstate) in 0.2% BSA/PBS over night at 4°C. After three washing steps with PBS, the specimen were incubated with the corresponding AlexaFluor® -labeled secondary antibodies for 30 min at room temperature. Finally, the slides were again washed three times with PBS (5 min), embedded in FluorSave^TM^ Reagent mounting medium and covered with 8 mm ×8 mm glass cover slips.

### Statistical Analysis

The number of independently performed experiments is stated in the respective figure legend. Bar graph data are mean values ± SEM. Statistical analysis was performed with the GraphPad Prism software version 3.03 (GraphPad Software, San Diego, CA, USA). For statistical comparison of groups one-way ANOVA followed by Dunnett post hoc test was used. Values of p<0.05 were considered statistically significant. Graph Pad Prism was used for curve fitting (nonlinear, sigmoidal dose response curves with a variable slope based on a Hill Emax model), and for calculation of the IC50 values for each compound.
